# Ownership of patient care: a behavioural definition and stepwise approach to diagnosing problems in trainees

**DOI:** 10.1007/s40037-013-0058-z

**Published:** 2013-04-23

**Authors:** Kimberly McLaren, Julie Lord, Suzanne B. Murray, Mitchell Levy, Paul Ciechanowski, Jesse Markman, Anna Ratzliff, Michael Grodesky, Deborah S. Cowley

**Affiliations:** 1 Department of Psychiatry and Behavioral Sciences, University of Washington, P.O. Box 354694, Seattle, WA 98195-4694 USA; 2 Department of Psychiatry and Behavioral Sciences, University of Washington, P.O. Box 356073, Seattle, WA 98195 USA; 3 Department of Psychiatry and Behavioral Sciences, University of Washington, P.O. Box 354694, Seattle, WA 98195 USA; 4 Department of Psychiatry and Behavioral Sciences, University of Washington, P.O. Box 356560, Seattle, WA 98195 USA; 5 Department of Medicine, General Internal Medicine, University of Washington, P.O. Box 354760, Seattle, WA 98105 USA

**Keywords:** Ownership, Supervision, Faculty development, Theory of planned behavior, Professional development

## Abstract

In medical education, behavioural definitions allow for more effective evaluation and supervision. Ownership of patient care is a complex area of trainee development that crosses multiple areas of evaluation and may lack clear behavioural definitions. In an effort to define ownership for educational purposes, the authors surveyed psychiatry teaching faculty and trainees about behaviours that would indicate that a physician is demonstrating ownership of patient care. Emerging themes were identified through analysis of narrative responses in this qualitative descriptive study. Forty-one faculty (54 %) and 29 trainees (52 %) responded. Both faculty and trainees identified seven core elements of ownership: advocacy, autonomy, commitment, communication, follow-through, knowledge and teamwork. These seven elements provide a consensus-derived behavioural definition that can be used to determine competency or identify deficits. The proposed two-step process enables supervisors to identify problematic ownership behaviours and determine whether there is a deficit of knowledge, skill or attitude. Further, the theory of planned behaviour is applied to better understand the relationship between attitudes, intentions and subsequent behaviour. By structuring the diagnosis of problems with ownership of patient care, supervisors are able to provide actionable feedback and intervention in a naturalistic setting. Three examples are presented to illustrate this stepwise process.

## Introduction

As graduate medical education evolves, teaching faculty are tasked with balancing supervision requirements with trainee autonomy, all the while ensuring patient safety and quality of care. Some educators are concerned that changes in the training environment, such as reduced duty hours and closer supervision of trainees, adversely affect the development of trainees’ ownership of patient care [1–4]. There is a growing literature describing the tension between adequate supervision and trainee autonomy, including the complexity of determining competence for independence in particular areas [5, 6], identifying entrustable professional activities [7–9] and novel approaches to increasing resident responsibility and ownership [10, 11]. Some professional activities, such as history-taking and procedural skills, lend themselves more naturally to systematic and objective evaluation for competence and progression to the next level of responsibility. Other aspects of medical training are more difficult to define, increasing the challenge for supervisors to determine competence, and perhaps more importantly, to identify deficits and create remediation plans.

‘Ownership’ is a broad term encompassing aspects of professionalism, patient care and patient safety. Graded responsibility is a cornerstone of medical education. Supervisors expect trainees to take increasing ownership of patient care as they progress through training. However, ownership involves a constellation of, often intangible, behaviours and attitudes. We may recognize a lack of ownership when we see it, but without clear behavioural definitions, it can be difficult to clearly articulate the deficit to the trainee. From discussions with our own faculty and with participants of a workshop at the American Association of Directors of Psychiatric Residency Training annual meeting [12], we understand that feedback about such deficits may be avoided because it seems too abstract or judgmental. This is not uncommon in the evaluation and feedback of professionalism topics.

Without clearly articulated concepts, educators have difficulty specifying trainees’ deficits and providing actionable feedback [13]. In a qualitative study of faculty attitudes about teaching professionalism in general, faculty perceived professionalism as intangible and difficult to articulate. Its definition was perceived to be fluid and influenced by local culture, medical speciality and task [14]. This amorphousness was perceived as a barrier to teaching.

In contrast, faculty development initiatives that provide explicit definitions and expectations for trainees and supervisors improve faculty skills, engagement in teaching and trainee professionalism ratings [13, 15]. Consequently, we believe that the core elements of ownership of patient care need to be behaviourally defined for teaching and assessment purposes.

Several of our psychiatry teaching faculty spontaneously raised concerns about trainee ownership of patient care. Because this term can refer to various aspects of doctoring (e.g., patient care and safety, professionalism), we believed it was important to determine which specific behaviours our faculty considered to be components of ownership. Clarifying the behaviours that define ownership was essential for design of specific, targeted educational interventions. We were also curious as to which behaviours trainees identified as indicating that a physician was taking ownership of patient care, and whether these were different than those identified by faculty. Based upon our survey of psychiatry faculty and trainees, we present a working definition of core behavioural elements of ownership. We then propose a method to diagnose deficits in a trainee’s ownership of patient care, in order to facilitate feedback and remediation in clinical settings.

## Methods

### Design

We used a qualitative descriptive approach with open-ended free text responses from faculty and trainees to assess opinions about which behaviours indicate that a physician is taking ownership of patient care.

### Sampling

In the fall of 2009, all teaching faculty (76) and general psychiatry resident trainees (56) from the University of Washington Department of Psychiatry and Behavioural Sciences were invited by email to respond anonymously to on-line open-ended questions. The questions were sent simultaneously to both groups.

### Procedure

The University of Washington Human Subjects Division determined that this study did not require submission or approval by that body.

Participants answered a structured, open-ended question regarding which behaviours would indicate that a physician is taking ownership of patient care.

### Analysis

The research team (seven faculty members) reviewed and coded the written texts for both the trainee and faculty responses. First, the team members individually open-coded the text data for trainee respondents. This was followed by a team meeting where individual codes were refined and synthesized. The entire process was repeated for the faculty respondents without specific reference to the codes used with the trainee analysis.

It was recognized that themes derived from the analysis of the trainee responses could not be fully bracketed from the research team during the analysis of the faculty responses. However, it was felt that approaching the two response groups separately would improve methodological rigor by allowing the team to not only be open to previously unmentioned themes, but also to reaffirm any similarities that emerged.

## Results

Forty-one faculty members (54 %) and 29 resident trainees (52 %) provided narrative comments. We derived emerging themes from both groups. While many themes were present in both faculty and trainee comments, a proportion were also reported exclusively by each group.

Common and divergent themes are presented in Table 1. Each identified element of ownership is described, using common phraseology from respondents, including representative quotes from faculty and trainees.

**Table 1:** Elements of ownership of patient care

Behavioural descriptors	Representative quotes
Identified by faculty and trainees	
** Advocacy** • Being the patient’s advocate • Being vocal/assertive about the patient’s best treatment/care • Challenging the team as needed	*‘…advocacy for the patient within the system; doing what’s right for them, even when it is a hassle’*
	*‘…advocate for the course of action they believe is in their patient’s best interest’*
**Autonomy** • Independence • Self-awareness (including own limitations) • Seeking consultation when needed • Thinking critically • Decision-making	*‘I am impressed when residents manage problems that arise on their own, or after asking for my input, rather than always turning things over to me’*
	*‘Feeling comfortable making autonomous decisions and using attending as a consultant, when advice is needed’*
	*‘Offer a plan to your attending for revision, but make sure you HAVE a plan and, if appropriate, a contingency plan’*
** Commitment** • Putting professional responsibilities first • Going the extra mile • Engagement • Actively participating • Being invested • Providing excellent care to patients seen on cross-cover or for a shift	*‘Ownership should not only apply to patients on your service, but also patients you are cross*-*covering for, e.g., on weekends. With an increase in hand*-*offs with more regular work hours, residents must learn to take ownership for patients they have temporary responsibility for’*
	*‘Not just to be medication prescriber but to be actively involved in all aspects of patient care such as disposition plan, follow up, talk with outpatient provider and family members’*
**Communication** • Communicating with patients, families, other providers regarding transitions of care	*‘Coordination of care, including effective and inclusive communication with all team members and family’*
	*‘Call families and outpatient providers’*
	*‘Providing up to date, comprehensive, and informative sign out’*
	*‘Physician is able to articulate goals of treatment/plans to patient’*
**Follow-through** • Being thorough, dependable, conscientious, diligent, responsive, accountable • Coordinating care • Taking care of detail • Carrying out the treatment plan • Making sure things do not fall through the cracks	*‘Physician follows up on labs/test/medications that he/she has ordered. Does not need to be reminded. Takes full responsibility for all aspects of care’*
	*‘YOU make sure that all the loose ends are tied together, tests have been ordered and results checked, treatment changes have been carried out, scheduled meetings have taken place, etc’*
	*‘Actively participates in the daily work necessary to enact treatment plan’*
**Knowledge** • Reading and learning all about the patient	*‘…knowledge of the patient’s presenting concerns, the goals for hospitalization, the status of these goals and what that resident/fellow needs to do as a team member to move the case forward’*
	*‘Knowing the labs and the past history, being generally up*-*to*-*date before rounds start, trying to do some patient*-*specific learning when the treatment plan isn’t cut and dried’*
	*‘Strives to know more about the patient than any other person on the team; the ‘go*-*to’ person for knowledge about the patient’*
**Teamwork** • Collaboration • Awareness of one’s role within the health care team • Taking ownership for the part of the care you are responsible for • Shared/team responsibility for the patient • Being considerate of the team in scheduling absences, other activities	*‘…a sense of shared primary responsibility for part of the care of a patient’*
	*‘Considers impact on ward/service functioning when scheduling elective absences (doctor appointments, supervision,* etc*.)’*
	*‘…facilitated the smooth functioning of the team …understood and respected my role as an attending, but this did not stop him from taking ownership of the team’*
	*‘Making collaborative decisions about the patient’*
	*‘Checking in with team members* – *i.e. overnight nursing staff’*
Identified by faculty only	
**Continuity of care** • Ensuring good care even when you are not there (e.g., sign-outs, handoffs)	*‘I am concerned that there is a process of ‘hand offs’ where each person hangs in there for their ‘shift’ hoping that nothing goes wrong … until they can hand off the patient to the next person in the queue’*
	*‘Ensuring continuity of care when you are absent from the service’*
**Excellence** • Providing high quality of care	*‘YOU have primary responsibility for the patient receiving high quality of care and having a good outcome’*
	*‘Making all efforts needed to deliver best care despite the time and individual sacrifices that may entail’*
**Extra time** • Spending extra time • Availability after regular hours • Being the patient’s doctor 24/7	*‘…coming in early to read about the patients and staying late to read the scientific literature’*
	*‘Tending to all the patients’ needs…after hours if needed’*
**Initiative** • Being proactive rather than passive	*‘It means being the patient’s doctor, in what I guess is the old*-*fashioned sense of that term: you are the one who calls to check on how they are doing, you are their contact person within the system, you take the initiative to involve their family members when appropriate…, you interact with the [social worker] to get referrals taken care of…, you call other services…, you make sure that they are scheduled back appropriately and that they have this information’*
	*‘…being thorough and proactive in patient care rather than waiting to have the attending tell them what to do’*
**Sense of vocation or calling** • Having a deeply personal sense of responsibility • Altruism • Sacrifice • Earning and being worthy of trust	*‘… the deep sense that I have an abiding duty to my patients that transcends incidentals of schedules, shifts, hours worked…’*
	*‘a sense of personal commitment’*
Identified by trainees only	
**Hierarchical tension** • Struggles with attendings regarding degree of autonomy/independence	*‘Taking an active part in discussions with the patient as opposed to just letting attendings decide what they want to do’*
	*‘To have the attending defer to you during morning rounds with the nurses’*
**Leading** • Being in charge	*‘Primary provider in discussions, management, and treatment decisions with the patient’*
	*‘To get paged for all issues from the nursing staff first’*

The responses contain a depth and richness which speak to the complexity of this topic, highlighting the strength of qualitative research methods for our purpose in this study. Responses indicate the range of privilege and responsibility in patient care. For example, the theme of autonomy includes the importance of thinking independently, generating a treatment plan and not relying on the attending (or another member of the treatment team) to make all of the decisions. However, it also includes an awareness of one’s limitations—knowing when to consult and ask for direction.

Many responses contain elements from several themes. For example, we felt the following quote best represented the theme of commitment: *‘Not just to be medication prescriber but to be actively involved in all aspects of patient care such as disposition plan, follow up, talk with outpatient provider and family members.’* Additional themes contained within this quote may include advocacy, communication, follow-through and teamwork.

## Discussion of qualitative study

While it is acknowledged that findings from qualitative methods are not usually considered to be generalizable, the strength of the method lies in the depth of the descriptions from the respondents.

The trainee and faculty groups both perceived advocacy, autonomy, commitment, communication, follow-through, knowledge and teamwork as core elements of ownership. We chose to focus on these themes as being central to our definition of ownership, since there was consensus. According to Holtman, social networks play an important role in defining and maintaining standards of professional behaviour. Within a network, ‘greater normative consensus (about professional behaviour) should produce greater consistency in informal feedback (and) fewer mixed messages’ [16]. The themes identified separately by faculty and trainees may well be important components of ownership, but may also be confounded by added programmatic bias, professional development, generational differences, etc.

These core elements of ownership were developed within a single residency programme in psychiatry, and thus may not be generalizable. Clearly, attitudes are informed by local culture, including speciality. Much of what is written in the surgical literature about ownership pertains to curtailed duty hours [2, 3, 17]. Other procedure-oriented specialities focus on entrustable activities [5, 8, 18]. Balancing autonomy and supervision is discussed across specialities [10, 11, 19, 20]. Elements such as knowledge of the patient, follow-through, communication, autonomy, and teamwork are also included in these discussions. While these studies eloquently highlight various aspects of ownership, our study attempts to provide a more comprehensive and inclusive definition of ownership.

There may be factors within our proposed themes that deserve further analysis. Future work could include measures such as replicating results and factor analysis, which may include focus groups to triangulate results. Particularly, faculty themes that include personal sacrifice, spending extra time and a sense of calling could be more thoroughly discussed in a focus group, allowing for questions about work-life balance and realistic expectations.

This is a pilot study with preliminary findings serving as a stimulus for additional investigation. Future studies repeating this survey in other specialities and at other institutions would increase generalizability. Additional data could be collected about respondents, such as years of training or years of supervisory experience, date of birth, and speciality. These data may help identify factors that play a role in individual definitions of ownership, particularly where faculty and trainee definitions diverge. Potential areas of additional study could include inpatient versus outpatient providers and interprofessional comparisons given the prevalence of team-based systems of care.

## Diagnosing ownership problems

The ultimate value of our definition is in its utility for supervising trainees in practice. While we may be able to recognize that a trainee is not ‘taking ownership,’ we may get stuck pinpointing the specific problematic behaviour. If we are able to verbalize the behaviour, we may have difficulty identifying the underlying deficit. Each of these can interfere with giving useful and actionable feedback. This definition provides a behavioural framework for supervisors (either supervising faculty or senior trainees in a supervisory role) to use for assessment and remediation. As trainees demonstrate an appropriate level of ownership, supervisors may begin to allow more autonomous practice, taking a more indirect role in patient care, while still assuring patient safety and quality of care. However, when trainees are identified as having problems taking ownership, a structured approach to identifying the specific deficit is warranted.

Remediating struggling trainees can be challenging, and we propose the application of our definition as a guide to diagnosing ownership deficits. Below, we describe a two-step process to identify specific areas for remediation.

Step I: Identify the target behaviour
  First, consider which element of ownership is most problematic: advocacy, autonomy, commitment, communication, follow-through, knowledge or teamwork. Table 1 may help guide supervisors in ‘naming the problem’—matching the concerning behaviour with an element of ownership. While deficits may overlap multiple domains, we suggest focusing on one or two areas most needing improvement, to avoid overwhelming the trainee and supervisor in the feedback session.
Step 2: Define the deficit After identifying the specific element of ownership requiring improvement, consider whether there is a deficit in knowledge, skills, or attitude.

*Knowledge*. Does the trainee know what is expected? Beginning trainees in particular may need to be educated or reminded about expectations regarding ownership of patient care.

*Skills*. Does the trainee have the necessary skills to carry out the desired behaviour? In assessing skills, it is useful to know about the trainee’s level of training, prior experience, and whether he or she has demonstrated the desired skills in the past. It is very useful to speak with the programme director or previous supervisors about whether the trainee has struggled on other rotations. Supervision can then focus on skill development, deliberate practice of the skill and ongoing formative feedback.

*Attitude*. Attitude deficits may be more difficult to define, and arguably most important, as professionalism is largely defined by attitudes. A trainee may have a pervasive underlying problem, such as psychiatric illness, substance abuse, burnout, or speciality dissatisfaction requiring residency-level support. Less pervasive problems may involve attitudes related to the specific rotation, supervision style or desired behaviour. To assess the latter, we propose using the theory of planned behaviour, a structured approach to attitudes and their consistency with one’s intentions and subsequent behaviours.

### Theory of planned behaviour in assessing ownership attitudes

Fishbein and Ajzen [21] described the relationship between attitudes and behaviour in the theory of reasoned action (TRA). According to the TRA, the best predictor of actual behaviour is intention to behave. Intention is influenced by (1) attitude towards the behaviour and its consequences and (2) subjective norms, including perceptions about others’ expectations and social pressure. The theory of planned behaviour (TPB) expanded on the TRA by including a third factor: (3) perceived control or difficulty in carrying out the behaviour [22, 23]. The additional influences of environmental constraints and individual skill and ability have also been described [24].

The TPB has been successfully applied to health-related behaviour, with respect to predicting both patient and provider behaviour. Examples include smoking cessation [25], condom use [26], healthcare worker glove use [27], and nurses’ pain management behaviour [28]. The TPB has also been suggested as a framework for assessing and teaching professionalism [29, 30].

By recognizing the various factors that influence intention to behave, thereby predicting likelihood of behaviour, we can more precisely assess the cause of undesirable behaviour or lack of desired behaviour. Likewise, we can structure educational interventions to target the specific deficit and foster a learning environment that increases likelihood of desirable behaviours being ingrained.

Applying the TPB to ownership of patient care would involve considering the following:

1. Does the trainee believe that ownership behaviours are meaningful and valuable for patient care?
2. Does the trainee believe that most physicians, and their peers, engage in ownership behaviours, and that such behaviours are important to be a good and well-respected trainee and/or physician?
3. How confident is the trainee that s/he can carry out ownership behaviours, given his/her own abilities and environmental constraints?

For example, a trainee’s perception that stated expectations do not represent the reality of practice and values of the programme (the ‘hidden curriculum’) decreases motivation and promotes cynicism. Thus, trainees may lack intrinsic conviction of the importance of a given behaviour. Alternatively, increases in attending oversight due to supervision rules and regulatory pressures may lead trainees to believe that their contribution is marginalized. Excessively close supervision (micro-management) may hinder ownership in an otherwise capable trainee [19]. Finally, an individual’s lack of confidence, in the absence of a skills deficit, can be addressed by encouragement and experience of success.

Case examples illustrating this two-step process to diagnose ownership problems are presented in Box 1.

**Box 1:** Case examples illustrating the assessment process

Case 1: Ann, a first-year trainee on her first rotation, has difficulty completing tasks assigned in rounds. As a first step, her supervisor identifies this as a problem with follow-through. When given feedback, Ann is embarrassed and says that she cannot keep track of everything that she needs to do. Her supervisor conceptualizes this as a skills deficit, and teaches Ann to keep a structured to-do list with boxes to check when she completes each task.
Case 2: Charlie is half way through training. He knows about his patients, completes assigned tasks, and gives knowledgeable answers to questions. However, he looks to the supervisor to interview patients, lead team meetings, and determine diagnoses and treatment plans. The supervisor identifies a deficit in autonomy, realizes that he has not specifically reviewed his expectations with Charlie, but learns that Charlie has shown more initiative on prior rotations. He suspects an attitude issue, but nevertheless reviews with Charlie his expectations that Charlie be the team leader. Using the theory of planned behaviour, he explores Charlie’s views of the importance, social norms around, and difficulty with taking on this role. Charlie states that he considers this goal meaningful, but that he has found it difficult and unimportant that he be more autonomous and serve as the team leader on this rotation, since the supervisor takes care of everything. The supervisor considers that he might be ‘micro-managing,’ which is interfering with Charlie’s ability to take ownership of patient care. Together, they make an action plan that Charlie will act as the team leader, with specific goals for what this entails. The supervisor agrees to allow Charlie to be more autonomous, while ensuring patient safety.
Case 3: Stephanie, early in her second year of training, is eager to take on responsibility for patients and ‘own’ patient care. She confidently gives her opinion of diagnoses and treatment plans, but considers a very narrow differential diagnosis and displays lack of appropriate knowledge or clinical reasoning to back up her diagnoses and plans. Her supervisor views her as an ‘over-owner’, taking on more ownership than warranted by her clinical skills. In this case, the supervisor’s feedback and guidance can acknowledge Stephanie’s high level of ownership and desire to take on responsibility, but focus on the need to develop other competencies (differential diagnosis, medical knowledge, responsiveness to constructive feedback) to enhance her clinical skills.

## Discussion

We suggest using this framework in the context of shared clinical work, including inpatient ward teams and outpatient clinics. Deficits can then be explored and remediated in ‘real time,’ through experiential learning, immediate feedback and naturalistic opportunities to practice new skills under direct observation.

By simplifying the process of identifying ownership deficits, we believe that faculty will be more likely to address observed behavioural deficits. Faculty and peers may avoid underperforming trainees for a variety of reasons. This resulting isolation increases risk for more unprofessional behaviours by reducing opportunities for social learning and normative social pressure [16]. Giving formative feedback helps supervisors stay engaged with underperforming trainees.

Future evaluation of this framework could occur at three levels: supervisors’ confidence in addressing ownership deficits, skill in applying the framework in clinical practice and downstream effects on trainee performance. We believe that increasing emphasis on teaching ownership, and developing tools for its evaluation, will also increase faculty self-awareness and promote good role modelling of ownership of patient care.

## Conclusions

Teaching faculty and many senior trainees are expected to provide supervision, often without specific education about being a supervisor. Difficult to define concepts, such as ownership, can seem ambiguous and ‘unteachable.’ However, behavioural definitions enable supervisors to better evaluate competence and identify deficits. Further, such definitions may translate into entrustable professional activities and inform handoffs of trainees from one supervisor to another.

The core elements of ownership discussed here were derived from both trainees and faculty, thus increasing likelihood of buy-in from both groups.

We propose a two-step process for assessing concerns about a trainee’s ownership of patient care, using seven core elements of ownership, together with consideration of knowledge, skills and attitudes, incorporating the TPB. We offer a specific and actionable framework for addressing this aspect of professional development.

## Essentials

- Behavioural definitions are necessary in order for supervisors to systematically and objectively evaluate trainees.
- A consensus-derived behavioural definition of ownership of patient care is presented and includes the following elements: advocacy, autonomy, commitment, communication, follow-through, knowledge and teamwork.
- A two-step process for identifying trainee deficits in ownership begins by determining which behaviour needs improvement and then considering whether the deficit is one of knowledge, skill or attitude.
- The TPB is applied to better understand the relationship between attitudes, intentions and subsequent behaviour.
- Deficits are best explored and remediated in the practice environment within the supervisory relationship.
